# Supplementary material for: Causal interplay between lactose intolerance and gut microbiota: a combined bidirectional Mendelian randomization and in vivo validation study
Source: Front Nutr. 2026 Jun 1;13:1803337. doi: 10.3389/fnut.2026.1803337 (PMC13265576; doi:10.3389/fnut.2026.1803337)
Supplement: Supplementary file 1 [file Data_Sheet_1.zip › supplementary materials/Forward/leave one out cross validation/ebi-a-GCST90027602.finngen_R12_E4_LACTONAS.pdf]

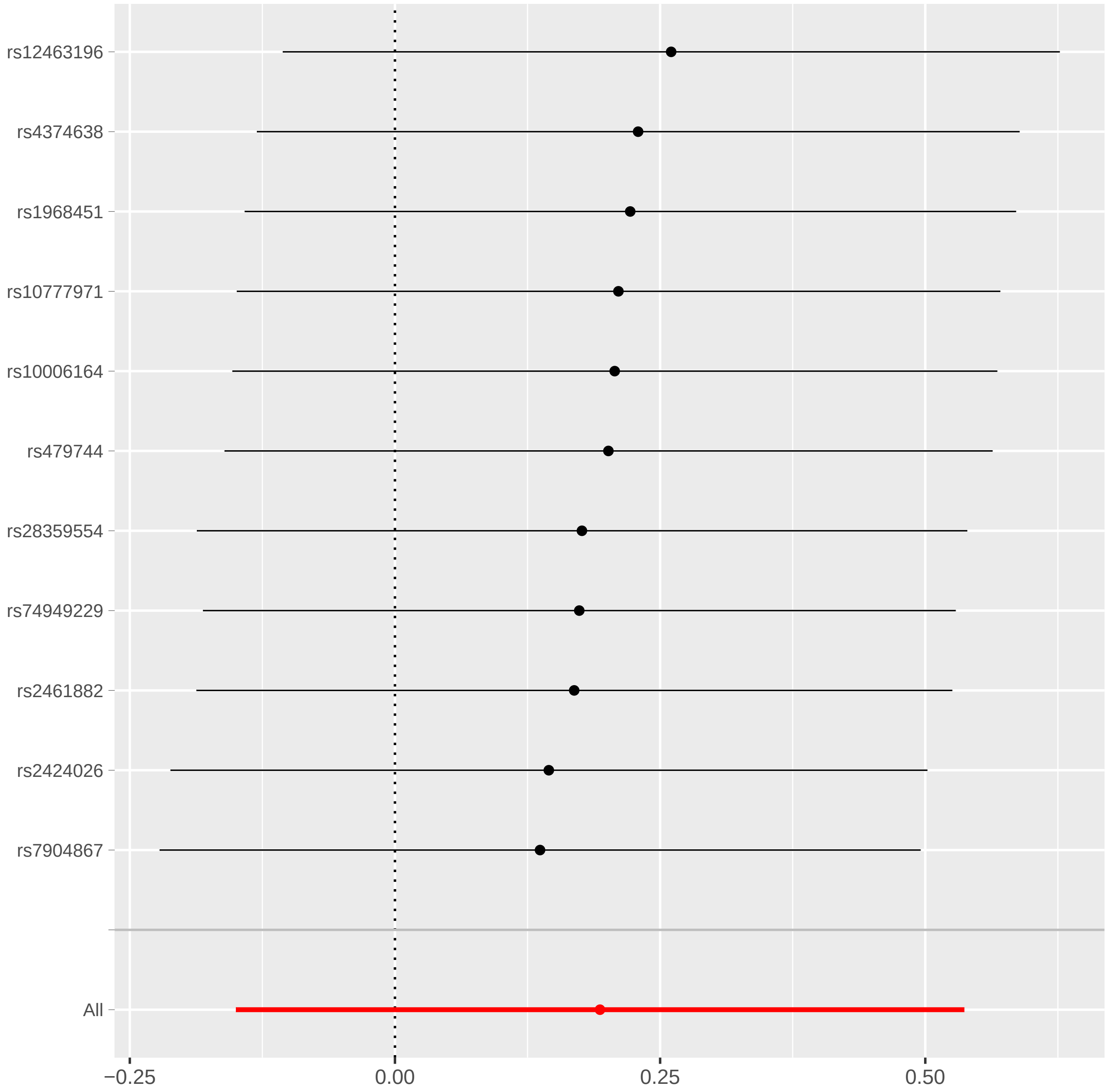

MR leave−one−out sensitivity analysis for
'Gut bacterial pathway abundance (PWY.6897..thiamin.salvage.II)' on 'Lactose intolerance, other/unspecified || id:finngen_R12_E4_LAC
